# Role of CD146 (MCAM) in Physiological and Pathological Angiogenesis—Contribution of New Antibodies for Therapy

**DOI:** 10.3390/biomedicines8120633

**Published:** 2020-12-19

**Authors:** Ahmad Joshkon, Xavier Heim, Cléa Dubrou, Richard Bachelier, Wael Traboulsi, Jimmy Stalin, Hussein Fayyad-Kazan, Bassam Badran, Alexandrine Foucault-Bertaud, Aurelie S. Leroyer, Nathalie Bardin, Marcel Blot-Chabaud

**Affiliations:** 1 Hematology Department, Center for CardioVascular and Nutrition Research C2VN, Faculty of Pharmacy, Timone Campus, Aix-Marseille University, Institut National de la Santé et de la Recherche Médicale (INSERM), Institut National de Recherche Pour L’agriculture, L’alimentation et L’environnement (INRAE), 13005 Marseille, France; xavier.heim@etu.univ-amu.fr (X.H.); clea.dubrou@univ-amu.fr (C.D.); richard.bachelier@inserm.fr (R.B.); wtraboulsi@gmail.com (W.T.); jimmy.stalin@unifr.ch (J.S.); alexandrine.bertaud@univ-amu.fr (A.F.-B.); aurelie.leroyer@univ-amu.fr (A.S.L.); nathalie.bardin@univ-amu.fr (N.B.); marcel.blot-chabaud@laposte.net (M.B.-C.); 2 Laboratory of Cancer Biology and Molecular Immunology, Faculty of Science, Lebanese University, Hadath 1104, Lebanon; hfayyadk@gmail.com (H.F.-K.); bassam.badran@ul.edu.lb (B.B.); 3 Service d’immunologie, Pôle de Biologie, Hôpital de la Conception, Assistance Publique Hôpitaux de Marseille (AP-HM), 13005 Marseille, France

**Keywords:** CD146, angiogenesis, inflammation, endothelial cell, cancer, monoclonal antibodies

## Abstract

The fundamental role of cell adhesion molecules in mediating various biological processes as angiogenesis has been well-documented. CD146, an adhesion molecule of the immunoglobulin superfamily, and its soluble form, constitute major players in both physiological and pathological angiogenesis. A growing body of evidence shows soluble CD146 to be significantly elevated in the serum or interstitial fluid of patients with pathologies related to deregulated angiogenesis, as autoimmune diseases, obstetric and ocular pathologies, and cancers. To block the undesirable effects of this molecule, therapeutic antibodies have been developed. Herein, we review the multifaceted functions of CD146 in physiological and pathological angiogenesis and summarize the interest of using monoclonal antibodies for therapeutic purposes.

## 1. Introduction

Unlike many other physiological processes that initiate and develop only during embryo implantation and fetal development [1], angiogenesis, which is characterized by the production of new blood vessels from pre-existing microvasculature, also occurs in the adulthood stage and is then referred to as neo-angiogenesis [2] (to simplify, the term “angiogenesis” will be used for both developmental stages in this review). Indeed, beside embryonic development, angiogenesis is involved in diverse processes like reproduction, renewing of damaged vessels, nurturing of organs after ischemia or strokes, wound healing, or tissue repair. Given these fundamental roles, it is obvious that multiple proteins exist to modulate angiogenesis but also vascular system development. In fact, angiogenesis is finely regulated by soluble factors including proangiogenic growth factors (as VEGF, b-FGF, HGF, etc and their receptors) and anti-angiogenic factors (as thrombospondin 1, angiostatin, endostatin, PF4, etc) in addition to insoluble molecules present in the extracellular matrix (as collagen, fibronectin, etc) [3]. The homeostatic balance between these soluble factors contributes to the onset and maintenance of physiological vascularization. Notably, endothelial cells migrate, proliferate, and differentiate into capillaries in response to a concentration gradient of pro-angiogenic growth factors [4]. Many diseases have been attributed to a deregulation of both angiogenic stimuli and inhibitors [5,6,7]. Indeed, an increase in angiogenic stimuli and a decrease in the angiogenic inhibitors constitute the hallmark of many cancers [8], cardiovascular disorders [9], and chronic inflammatory diseases [10], leading to abnormal neovascularization. Along this line, the cell adhesion molecule of the mucin family, CD146, appears to be expressed at the endothelial junction but also at the apical membrane of endothelial cells where it was recently found to act as a co-receptor for the key angiogenic receptor Flk-1 (VEGFR-2) [11]. Indeed, CD146 is expressed on endothelial cells, smooth muscle cells, and pericytes, and thus on the entire vessel [12]. This membrane glycoprotein is also found as a circulating soluble form which displays multifaceted effects on endothelial and surrounding cells [13]. 

A huge body of evidence, not limited to mammals, highlights the significance of CD146 in angiogenesis and vascularization. Thus, the aim of this review is to summarize the role of CD146/soluble CD146 in physiological and pathological angiogenesis and shed light on the therapeutic approaches that have been so far developed to fight their adverse effects.

## 2. CD146: Generalities 

CD146, also referred to as melanoma cell adhesion molecule (MCAM), hemopoietic cell adhesion molecule (HEMCAM), MUC18, S-Endo1, or A32 antigen, is a cell adhesion molecule essentially expressed on the entire vascular tree that belongs to the immunoglobulin superfamily [14]. It plays a significant role in regulating vascular permeability, cell-cell cohesion, leukocyte transmigration, and angiogenesis [15,16,17]. The extracellular domain of this single-pass membrane glycoprotein is composed of two variable regions (V) and three constant regions (C2) V-V-C2-C2-C2, while the intracellular domain is relatively short, containing a single tyrosine residue that may become phosphorylated [18,19]. Two membrane isoforms of CD146 exist, short and long, generated by alternative splicing of the transcript in exon 15, leading to a shift of the reading frame. Despite expressing identical extracellular and transmembrane domains, these two isoforms differ by their cytoplasmic tail. The short isoform (shCD146) displays a shorter cytoplasmic domain encompassing one phosphorylation site for protein kinase C (PKC) and an interaction site with proteins containing a PDZ domain. In contrast, the long isoform (lgCD146) displays two phosphorylation sites by PKC and a dileucine motif for protein targeting to the basolateral membrane [18,20]. Of interest, the expression of these isoforms is spatially selective. The long isoform is located at the cell junction and is involved in structural functions while the short isoform is essentially expressed at the apical membrane of the cell and contributes to angiogenesis. [18,21]. Additionally, shedding of membrane CD146 proteins, as induced by matrix metalloproteinases, generates a soluble form (sCD146) that is detected in the sera of healthy people at a concentration around 260 ± 60 ng/mL [22]. Of interest, CD146 is conserved among species, suggesting its evolutionary significance for physiological development.

### 2.1. CD146 Expression Pattern and Functions

CD146 is expressed all along the vascular tree regardless of the vessel size and anatomical location, including endothelial cells, smooth muscle cells, and pericytes [23]. This distribution pattern is important for maintaining vessel architecture through heterotypic interaction among these cells via CD146 and its binding partners. As mentioned earlier, the long and the short membrane isoforms have different localizations on endothelial cells. lgCD146 is mainly stored intracellularly when the cells are not confluent. However, at confluency, lgCD146 is redistributed to inter-cellular junctions, outside the tight or adherens junctions, and regulate cell–cell cohesion, paracellular permeability, and monocyte transmigration. shCD146 is involved in regulating endothelial cells adhesion, migration, proliferation, and consequently angiogenesis [21]. CD146 was first identified on melanoma cells as a poor prognostic marker correlating with disease progression, but was later found to be expressed on various cancer cell lines such as breast, kidney, gastric, ovarian, and prostate cancers [14,20,24]. The mechanisms underlying CD146 upregulation on cancer cells remain to be found. Elevated membrane CD146 expression and high soluble CD146 concentration in plasma are associated with increased cancer cell proliferation, motility, metastatic dissemination, and tumor angiogenesis, along with a decrease in patients’ overall survival [25]. Moreover, CD146 is also expressed on several immune cell subsets, in particular on T lymphocytes [26,27]. Along this line, it was demonstrated that soluble factors in the tumor microenvironment induce CD146 expression on tumor infiltrating T lymphocytes. Indeed, the density of CD146 expressed on tumor-infiltrating CD4+ T cells is higher than that on peripheral T cells [26,28]. Analysis of the RNA profile of CD4+ CD146+ T cells from peripheral blood shows high levels of genes associated with Th17 cells (IL-17A, ROR-γ, IL-22, IL-26, IL-23R, CXCL-13, IL1-β, GM-CSF) which exacerbate inflammatory reactions and indirectly promote tumor progression [27]. In addition, Th17 cells contribute to auto-immune diseases progression as in multiple sclerosis (MS) and systemic sclerosis (SS). In fact, CD146+Th17 cells constitute the principal T-cell subset in the cerebrospinal fluid of MS patients and is considered as a poor prognostic marker [29]. CD146 was also found to be expressed on the intermediate and extra-villous trophoblasts [30,31].

### 2.2. CD146 Ligands and Signaling

Initially considered as an orphan receptor, successive studies have identified new ligands for CD146 (Table 1). Most of the newly discovered ligands emphasize the role of CD146 in angiogenesis, as these ligands were found to promote angiogenic effects on endothelial cells. For example, in one study, authors showed that netrin-1, a neuronal guidance molecule, induces human umbilical vein endothelial cells (HUVEC) proliferation, migration, and tube formation by interacting with CD146 but not VEGFR2 [32]. They also showed that netrin-1 binds to the domain IV of CD146 to induce activation of P38 and Erk1/2, a characteristic signaling pathway implicated in VEGFR2 signaling, and relied this effect to the fact that CD146 also acts as a co-receptor for VEGFR2. Indeed, siRNA experiment targeting CD146 abolished the angiogenic effects of netrin-1 on HUVECs while the knock-down of VEGFR2 barely induced similar results. In the same study, the authors used a monoclonal anti-CD146 antibody, AA98, which blocks CD146. They showed that netrin-1 lost its angiogenic effects after treating HUVECs with AA98. Consistently, AA98 efficiently reduced the number of blood vessels in Matrigel plugs conditioned with netrin-1 and grafted in mice, when compared to the group treated with netrin-1 without the antibody. Likewise, in a zebrafish embryo model, antisense morpholino oligonucleotides (MO) targeting CD146 inhibited netrin-1-induced angiogenesis and blocked the development of parachordal vessels. These data underscore the relevance of CD146 as a potent angiogenic molecule both in vitro and in vivo and highlight the capability of CD146 to mediate angiogenic signals even in the absence of conventional pro-angiogenic molecules like VEGF or b-FGF. 

Galectins are a family of soluble carbohydrate-binding lectins which mediate cell-to-cell and cell-to-ECM adhesions. As glycosylation accounts for nearly 35% of CD146 apparent molecular weight, it was tempting to speculate that CD146 could interact with sugar-binding proteins such as lectins. In particular, galectin-1, a protein produced by vascular, interstitial, epithelial, and immune cells, was found to interact readily with N-linked oligosaccharides of membrane CD146 and signal to protect endothelial cells from apoptosis as well as to regulate angiogenesis [33]. In this study, Jouve et al. evidenced that CD146 is mainly N-glycosylated and validated its interaction with galectin-1. They showed that galectin-1, by binding CD146, was protecting endothelial cells from apoptosis. In addition, in vivo experiments in zebrafish showed impaired vascular network formation and poorly developed intersomitic vessels upon knocking down galectin-1 [34] and CD146 [16], respectively. Moreover, in a galectin-1 knockout (KO) mouse model [34], tumor growth was markedly impaired and this effect was attributed to a weak tumor angiogenesis. In the same way, administration of anti-CD146 monoclonal antibody AA98 potently reduced tumor vessel formation in nude mice xenografted with human tumor cells (SMMC7721, SK-LMS-1, SW1990). Another study by Thijssen et al. [35] has shown that galectin-1 is significantly upregulated on activated endothelium and positively correlates with strong angiogenesis by augmenting VEGF receptors and transforming protein p21 (GTPase H-RAS) signaling and phosphorylation. Thus, galectin-1 effect on angiogenesis could be, at least in part, mediated by CD146, which is indeed a coreceptor for VEGFR2. Importantly, it was recently described that endothelial CD146 binds platelet-derived growth factor receptor-β (PDGFR-β) on pericyte and regulates the PDGF-induced activation of PDGFR-β signaling [36]. By this mechanism, CD146 appears to have a crucial role in recruiting adjacent pericytes in the endothelium, and hence, stabilize the developing vessels. All other ligands that are documented in the literature to interact directly with CD146 are summarized in Table 1 along with the consequent biological significance. 

Concerning sCD146, its binding partners are still largely unknown. In fact, angiomotin was described to be among the first binding partners of sCD146 on endothelial cells. Stalin et al. [22] showed that the interaction between sCD146 and angiomotin on endothelial cells induces cell proliferation, migration, and pseudo-capillary formation in Matrigel. This interaction activates angiogenesis and exerts a competitive inhibitory effect on angiostatin. Recently, integrin αvβ1 has been reported to interact with sCD146, an interaction that appears to be important in regulating blood brain barrier (BBB) permeability [37].

### 2.3. CD146 Mechanism of Action 

CD146 is mainly a monomeric protein. However, it has been shown to dimerize and multimerize in response to physiological stimuli [46]. Indeed, the stimulation of endothelial cells with VEGF or by means of anti-CD146 AA98 antibody results in CD146 dimerization as revealed by fluorescence resonance energy transfer (FRET) technology. This dimerization leads to conformational changes in CD146 structure and induces changes in ligand binding. However, whether this dimerization causes receptor auto-activation and -phosphorylation is not elucidated yet. 

**Figure 1:**
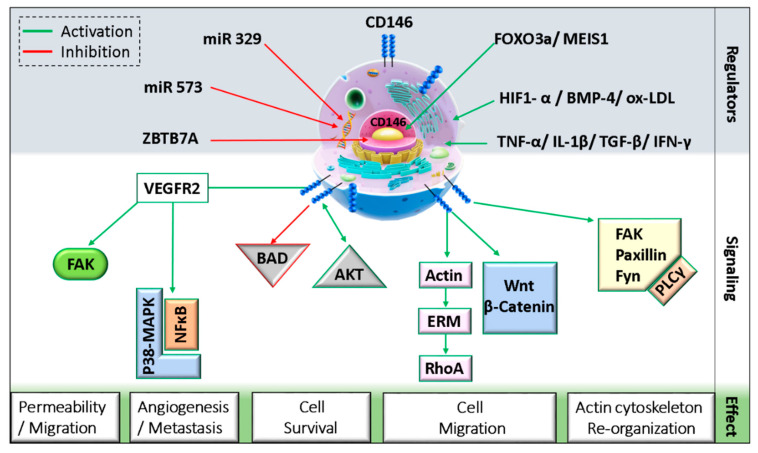
Schematic representation of CD146 cellular localization and regulation.(MEIS1: myeloid ecotropic viral integration site 1; HIF1-α: hypoxia-inducible factor 1 alpha; TGF-β: transforming growth factor-beta; IFN-γ: interferon-gamma; BMP-4: bone morphogenetic protein 4; ox-LDL: oxidized low density lipoprotein; ZBTB7A: zinc finger and BTB domain-containing protein 7A; FAK: focal adhesion kinase; BAD: Bcl-2 associated agonist of cell death; ERM: ezrin, radixin, and moesin; PLC-γ: phospholipase C-gamma).

CD146 can interact with diverse ligands that mediate and alter endothelial functions (Figure 1). In fact, CD146 activation induces the phosphorylation of p125^FAK^ and paxillin along with p59^fyn^ recruitment in cultured endothelial cells, leading to actin cytoskeleton reorganization and activation of transcription factors that modulate cell migration and survival [47]. Moreover, the intracellular domain of CD146 interacts physically with actin linked proteins of the ezrin-radixin-moesin (ERM) family, bringing them to the level of membrane protrusions. This CD146-mediated formation of microvilli like extensions will then allow the activation of RhoA by sequestering Rho guanine nucleotide dissociation inhibitory factor 1 (RhoGDI1), leading to an increase in cell motility. In addition, the stimulation of melanoma cells with Wnt5a induces CD146 redistribution within polarized structures known as W-RAMP (Wnt5a-mediated receptor-actin-myosin polarity), leading to membrane retraction and cell migration in a RhoA-mediated mechanism [48]. Finally, a defect in CD146 expression is associated with an upregulation of the canonical Wnt pathway and a downregulation of the non-canonical pathway [49]. 

On endothelial cells, both VEGFR2 and CD146 extracellular domains were found to interact physically even without VEGF binding [11]. A study showed that CD146 dimerizes upon VEGF-A stimulation in a mechanism involving RAC1, Nox4, and ROS production [50]. The inhibition of CD146 using siRNAs, miRNAs, or inhibitory antibodies as AA98 decreased VEGF-A-induced VEGFR2, p38/MAPK, and AKT phosphorylation and reduced NFκB activation in HUVECs [11]. Similarly, in lung vascular endothelial cells from CD146KO mice, VEGF-A stimulation resulted in decreased VEGFR2 and FAK phosphorylation [51]. Additionally, by regulating further junctional proteins such as VE-cadherin and PECAM, CD146 mediates VEGF-induced permeability in endothelial cells. 

As mentioned earlier, CD146 can interact with netrin-1 and mediate angiogenesis [32]. It has been shown in zebrafish that CD146 silencing inhibits netrin-1-induced vascularization. However, netrin-1 does not always stimulate angiogenesis. The controversy about its angiogenic role is attributed to the binding partner, as netrin-1 preferentially binds CD146 at low concentrations but UNC5B at high concentrations. Indeed, netrin-1 has higher affinity for CD146 as compared to its cognate ligand UNC5B. By binding UNC5B, signals will be triggered to counteract those of CD146 and thus inhibit angiogenesis [32]. On the other hand, netrin-1 can induce CD146 dimerization and VEGFR2 phosphorylation, and the inhibition of CD146 decreases netrin-1 effect on HUVEC migration, proliferation, and tube formation in vitro by reducing VEGFR2, ERK1/2, and p38 activation. 

Soluble CD146 is also involved in activating several signaling pathways. Stalin et al. showed that soluble CD146 binds angiomotin P80 on endothelial progenitor cells and HUVECs and regulates vessel formation and cell migration [22]. Mechanistically, soluble CD146 triggers a signalosome constituted of angiomotin, short CD146, VEGFR1, VEGFR2, and presenilin-1 in lipid rafts and phosphorylates VEGF receptors 1 and 2 on endothelial cells [52]. Within this signalosome, soluble CD146 promotes the proteolytic cleavage of short CD146 extracellular domain through matrix metalloproteinases/a disintegrin and metalloproteinase (MMP/ADAM), followed by intracellular cleavage of the cytoplasmic domain by presenilin-1. The generated so-called short CD146 intracellular fragment (shCD146 IC) will be then translocated to the nucleus where it associates with the transcription factor CSL to regulate the transcription of FADD, Bcl-xl, and eNOS genes that are involved in cell survival and angiogenesis [52].

Soluble CD146 also significantly contributes to tumor progression in different manners. This form, by associating with its cognate binding partners on cancer cells, activates the oncogene c-myc, which in turn activates signaling pathways leading to increased cell proliferation and migration [13]. 

## 3. CD146 in Physiological Angiogenesis

### 3.1. CD146 in Embryonic Angiogenesis 

The first direct evidence highlighting the significance of CD146 in physiological angiogenesis came from in vivo experiments in zebrafish [53]. Gicerin, the chicken homologue of human CD146, was overexpressed in zebrafish embryos to assess its impact on vascular development. Indeed, multiple gicerin constructs were designed to code for either the full-length receptor, the extracellular domain, the cytoplasmic domain, or constructs devoid of cytoplasmic domain. These experiments showed that mRNA encoding the extracellular domain of gicerin or gicerin lacking the cytoplasmic domain abrogate intersegmental vessels (ISVs) sprouting and inhibit angiogenic vessels development as confirmed by microangiography 55 hours’ post-fertilization. Constructs solely encoding immunoglobulin domain I of gicerin’s extracellular segment led to vascular anomalies in the embryos. At variance, overexpression of domains II to IV did not impact vascularization. Thus, the first immunoglobulin domain of gicerin is highly relevant for efficient angiogenesis in zebrafish. Similarly, knocking-down gicerin in zebrafish embryos using antisense morpholino oligonucleotide that disrupts protein translation resulted in anomalies in blood circulation 48 hours’ post-fertilization but also abnormal phenotype linked to pericardial edema. Alongside, microangiography in gicerin morphants revealed incomplete formation of ISVs with no sprouting, while the in situ hybridization technique validated the absence of vascular markers as kdrl and fli1a in the ISVs 24 hours post-fertilization without any detectable changes in VEGFR2 expression. Of interest, both the hemangioblast and angioblast specific markers, scl and fli-1a, were not altered in the gicerin morphants, suggesting that gicerin selectively inhibits angiogenesis with no effects on vasculogenesis. Expectedly, the angiogenic defect in gicerin morphants was rescued upon injecting synthetic gicerin mRNA. Concomitant with these results, gicerin was also found to be essential for VEGF-A signaling. In fact, induced expression of VEGF-A in zebrafish embryos resulted in ectopic vessel formation in common cardinal vein (CCV), a phenomenon that was abolished when embryos received gicerin-morpholino together with VEGF-A mRNA. This suggests that gicerin is indispensable for VEGF-A-induced angiogenesis. In another study by Chan et al. [17], CD146 was found to have a critical role in ISV development specifically by modulating the lumen size in blood vessels. Intriguingly, whole mount in situ hybridization experiments in 24 h-old zebrafish embryos revealed the localization of CD146 in the dorsal aorta, caudal artery, caudal vein, and the sprouting ISVs, thereby highlighting the fundamental role of CD146 in vascular development. In accordance with the aforementioned results, disrupting CD146 protein sequence using morpholino oligonucleotide) that deletes the amino-terminus region (V32 to T57) or totally abrogates protein expression resulted in a circulation shunt that led to partially bypass the caudal artery-vein system. Moreover, microangiography revealed that while both control and CD146 morphants expressed similar ISVs density, the vessels were narrower in the latter and the blood circulation was reduced. Moreover, Halt et al. elucidated the function of CD146 in mouse embryonic kidney development ex vivo [54]. This study unraveled a heterogenous population of endothelial cells in the embryonic kidney that variably stained for the markers CD31, VEGFR2, and CD146. Of interest, despite depleting CD31+ ECs from embryonic kidneys, regeneration of the endothelial cell network took place. On the contrary, the depletion of CD146+ ECs abolished this phenomenon. Additionally, it was found that CD146+/CD31- ECs potently regenerate a pool of CD31+ ECs following their depletion which then incorporate into the developing vessels of the embryonic kidneys. Hence, CD146+/CD31- cells exhibit a pivotal role during embryonic kidney development as they prove to act as progenitors for CD31+ ECs. From a therapeutic perspective, these findings raise the interest of using CD146+ EC progenitors in the treatment of endothelial injury in adult kidneys owing to their fundamental role in maintaining and stabilizing renal vasculature. In general, CD146 appears to be an indispensable factor in vascular development and structure (lumen size, morphology) by mediating a series of cellular events that ultimately lead to dynamic changes in the cytoskeleton during development. This conclusion is strongly supported by the fact that CD146 interacts with actin cytoskeleton in HUVECs and modulates the expression of several integrins of the β1 family on these cells [55]. Several studies have emphasized that sCD146 concentration is significantly increased in sera of patients with chronic kidney failure compared to healthy controls [56]. Concomitantly, serum sCD146 was found to be elevated in patients with diabetic nephropathy with a gradual increase in this concentration relative to the stages of the disease (stage IIIb: 558.6 ± 87.5, stage IV: 519.3 ± 80.8, stage V: 619.7 ± 32.5 versus control: 268.0 ±45.0 ng/mL), a phenomenon which is also seen in patients with chronic renal failure. Indeed, sCD146 is a hallmark of endothelial damage, explaining the positive correlation between its serum concentration and disease progression. 

### 3.2. CD146 in Pregnancy

The sustainability of many animal species is guaranteed by sexual reproduction. During this process, genetic material carried by a motile gamete fuses with that of a stationary gamete to give rise to a zygote. Afterwards, cells undergo mitotic divisions to form a blastocyst which will be implanted into the endometrium of the uterine wall. In fact, implantation is considered to be the most critical step in pregnancy and any factor perturbing this phenomenon will ultimately leads to miscarriage. Recently, several studies have addressed the physiological role of CD146 for successful pregnancies. For instance, Liu et al. have shown that CD146 is uniquely upregulated on both receptive maternal uteri and invasive embryonic trophoblasts during early stages of pregnancy but not on the non-pregnant uterus [30]. Indeed, a harmonized cascade of events occurs prior to blastocyst implantation. This includes a potent increase in the expression of adhesion molecules on the receptive endometrium, like L-selectin and integrins, which facilitate embryo implantation. Meanwhile, embryonic trophoblasts secrete matrix metalloproteinases and upregulate the expression of several adhesion molecules (VE-cadherin, α4β1) to enhance their invasive capabilities for reaching the spiral arteries. Of interest, a positive correlation exists between trophoblastic invasive potential and CD146 expression. It was found that CD146 is preferentially expressed on invasive trophoblasts, intermediate trophoblasts, but absent on the non-invasive ones, cytotrophoblasts and syncytiotrophoblasts [31]. In accordance, women suffering from pre-eclampsia expressed significantly lower CD146 levels on intermediate trophoblasts which hinders their invasive capabilities. Noteworthy, CD146 expression on the endometrium was found to be stringently specific and temporal; highly upregulated in early stages of pregnancy but totally lost afterwards and mostly absent in non-pregnant uterine endothelium [30]. Moreover, in vitro experiments using the antibody AA98 revealed a potent decrease in trophoblast attachment to uterine endothelial cells along with remarkable reduction in trophoblast outgrowth as compared to isotype matched mouse IgG-treated cells, hence validating the significance of CD146 in mediating proper embryo implantation. Similarly, in vivo administration of blocking anti-CD146, AA98, into the left uterine horn of pregnant mice (3.5 days post coitum) resulted in no or few poorly developed embryos while well-developed embryos were found in the right uterine horn that received isotype-matched control IgG [30]. Additionally, immunohistochemical analysis using anti-CD31 validated the poor neovascularization in the AA98-treated uterine horn, which suggests and reinforces the fundamental role of CD146 not only in early embryonic attachment and development but also in inducing adequate neovascularization leading to a successful pregnancy. Of note, CD146KO mice were found to be fertile, which suggests that other adhesion molecules may functionally compensate the loss of CD146 in vivo or some of CD146 functions may be operated, at least in part, by some other protein(s).

Of putative clinical importance, sCD146 is regarded as an important factor during pregnancy and is detected in sera of pregnant women. Its concentration fluctuates according to the embryonic developmental stage and can reflect several pregnancy-associated malignancies [57]. Notably, sCD146 is potentially a biomarker for selecting the optimal embryos during in vitro fertilization (IVF). In fact, Bardin et al. have shown that the concentration of sCD146 was elevated in the culture supernatants from blastocysts that failed to implant into the uterine wall while those that successfully embedded into the endometrium produced significantly lower amounts (1310 vs. 845 pg. mL^−1^) [58]. Given the bright expression of CD146 on early embryonic blastocyst and building on the aforementioned data, it is tempting to speculate that the increase of sCD146 in the supernatant of the in vitro fertilized embryos is the result of MMPs-mediated shedding of membrane CD146. This will decrease CD146 surface expression and subsequently diminish the potential of a successful uterine implantation. Thus, high membrane CD146 and low concentration of sCD146 contribute to an effective embryonic attachment, placental vascularization, and embryonic development. This could thus represent an innovative method for selecting embryos during in vitro fertilization.

### 3.3. CD146 in Adult Angiogenesis

Angiogenesis is a vital physiological process that ensures adequate delivery of blood and nutrients to all body parts. Several studies have highlighted the central role of CD146 in the development of the vascular system and other postnatal biological processes. Postnatally, the expression of CD146 becomes stringently regulated and shows tissue selectivity [23]. Wang et al. have shown that the physiological expression of CD146 is restricted to limited normal tissues in adults and that its adhesive strength is relatively weak as compared to other cell adhesion molecules [59]. CD146 expression was found to be spatiotemporally regulated, increased under circumstances, such as inflammation for a period of time, and then returned back to its basal expression levels. Indeed, the alternating mode of expression of CD146 in adults is not a random process, but on the contrary, finely tuned by various factors in response to body changes and stimuli. Unlike the prenatal life, species are more exposed to environmental and developmental changes postnatally. In fact, one of the mechanisms by which the body adapts to changes is by modifying the expression of adhesion molecules as CD146. For instance, from the onset of puberty and menstrual cycles in females, CD146 expression on the endometrial endothelial cells becomes elevated, which in turn sensitizes the cells to angiogenic stimuli, thus facilitating the repair phase [60]. Investigations focusing on the role of CD146 in organogenesis revealed its significance in kidney, liver, and retina development and vascularization [54,61]. Moreover, rapid proliferation of cells, a phenomenon typically occurring during wound healing and normal body growth, is characterized by a robust increase in CD146 expression which allows active interaction between cells themselves as well as with the surrounding microenvironmental structures. By doing so, CD146 helps transmitting signals inside the cells to accordingly modify their proliferative, migrative, and invasive potentials. Simultaneously, CD146 mediates and regulates a series of events leading to angiogenic responses. In particular, CD146 activates NF-κB in endothelial cells, a feature relevant to tip stalk-cell selection [62]. In the same way, sCD146 was shown to be indispensable in the vascular repair mechanism and to be an inducer of post-ischemic neovascularization [63]. 

## 4. CD146 in Pathological Angiogenesis 

### 4.1. CD146 in Miscarriage 

Approximately 10 to 15% of fertile women experience unexplained pregnancy loss, of which 5% suffer from consecutive spontaneous abortion [64]. The first evidence highlighting the implication of CD146 and its soluble form in pregnancy complications, precisely miscarriage, came from a study initiated by Pasquier et al. In this clinical study involving one hundred women with unexplained pregnancy loss versus 100 age-matched control women, the authors revealed that sCD146 plasma concentration significantly correlates with cases of pregnancy loss [57]. Indeed, the plasma concentration of sCD146 was significantly elevated in patients as compared to healthy subjects (279.2 ± 61.2 vs. 241.1 ± 46.7 ng/mL). Of interest, sCD146 plasma concentration was shown to be potently upregulated in the group of patients suffering from early pregnancy loss upon stratifying patients according to the gestation time at which spontaneous abortion occurs. The increase in sCD146 parallels endothelial damages and vascular anomalies. This result is in accordance with other studies demonstrating sCD146 as a molecule impeding trophoblast invasiveness and consequently placenta vascular development. For instance, sCD146 inhibited extravillous trophoblast (EVT) outgrowth and tube like-structure formation in vitro by negatively regulating their migratory and invasive properties [65]. In humans, these results are reflected by downregulation of sCD146 plasma concentration during physiological pregnancy, hence allowing trophoblasts to invade the endometrium to reach the spiral arteries. A study on 50 pregnant women also showed that sCD146 levels decrease in plasma as compared to non-gestational women. Indeed, repetitive systemic injection of recombinant sCD146 into pregnant rats reduced fertility and hampered embryos implantation by inhibiting glycogen cell (analogous to human cytotrophoblast) migration into the decidua. Alongside, another study showed that CD146 is strictly required during the implantation window (time at which the blastocyst adheres to the uterine wall) where its expression robustly increases on both the endometrium and the extravillous trophoblasts after which it starts to progressively faint [66]. Undoubtedly, abnormal expression of CD146 during this critical period or external interference with CD146 expression using blocking antibodies such as AA98 led to either fetus anomalies due to irregular vascularization or pregnancy miscarriage. In view of these results, sCD146 can be considered as a novel biomarker to predict obstetric anomalies and endometrial vascular development problems.

### 4.2. CD146 in Ocular Diseases

The retina is regarded as a physiological model for studying and analyzing angiogenesis. It is considered as an accessible organ relative to other parts of the body, and given its well-organized vascular anatomy, multiple animal models have been developed to study different vascular anomalies associated with ocular diseases. In fact, the retina is well compartmentalized into vascular and avascular regions as the human central retina is devoid of vessels. This characteristic mode of vascularization is extremely important for preserving healthy vision. In fact, aberrant retinal angiogenesis is linked to several retinal diseases that ultimately lead to blindness, such as retinopathy of prematurity, diabetic retinopathy, and age-related macular degeneration (AMD). Recently, it has been evidenced that oxygen is a key factor regulating retinal angiogenesis and vessel maturity [67]. Indeed, under physiological hypoxic conditions created during late embryogenesis as a result of increased cell differentiation and metabolic activity, HIF1-α becomes active, which in turn induces VEGF expression. This temporal expression of VEGF enhances angiogenesis in the retina. Intriguingly, CD146 gene expression was also found to be activated by HIF1-α. Further, since CD146 acts as a co-receptor for VEGFR2, it is tempting to speculate that VEGF and CD146, both HIF1-α inducible factors, act in a coordinated manner to regulate angiogenic responses and stabilize newly formed vessels in the retina. In pathological conditions, the increase in CD146 expression is associated with increased angiogenic responses which ultimately extend vascularization to the retinal avascular regions and consequently cause vision anomalies. Strikingly, sCD146 was found to act as a biomarker in exudative age-related macular degeneration (AMD), a chronic disease that progressively leads to irreversible loss of central vision in elderly people [61]. In this study, Liu et al. showed that the plasma level of sCD146 was significantly higher in 88 AMD patients in comparison with sex- and age-matched healthy controls (171.88 ± 65 vs. 125.52 ± 61 ng/mL). In fact, when AMD patients were further sub-categorized into patients with classic choroidal neovascularization (CNV) or occult CNV, the serum concentration of sCD146 was found to be significantly higher in the former cases than the latter. Likewise, the authors discovered that the soluble form of VEGFR2 was also significantly increased in the sera of AMD patients as compared to healthy subjects. Indeed, multiple clinical studies unveiled the role of VEGFR2 in the progression of exudative AMD and correlated it to the disease pathogenesis. Of notice, Harhouri et al. succeeded to show that sCD146 acts on endogenous endothelial cells to promote the expression of VEGFR2 and VEGF by these cells. They showed that both sCD146 and VEGF synergistically induce endothelial progenitor cell recruitment, proliferation, migration, and vascular-like structure formation in Matrigel plugs [63]. These results present sCD146 as a novel biomarker in various pathological diseases not limited to exudative AMD. As the secretion of sCD146 becomes relevant upon vascular damage, and since most of the clinical pathologies are manifested by vascular malformations, it would be of relevance to use a reliable biological marker that is easily quantifiable in human biopsies. Herein, we and others have shown that sCD146 represents an appealing biomarker for early detection of various diseases but also in other physiological processes. 

### 4.3. CD146 in Skin and Autoimmune Pathologies

The expression of CD146 has been identified on primary cultures of keratinocytes but was absent on healthy epidermis [68]. Indeed, CD146 increases in response to different skin pathologies such as psoriasis, Kaposi’s sarcomas, lichen planus, in the epidermis covering skin neoplasm, or in chronic or acute dermatitis. Of remark, Bardin et al. discovered that TNF-α, a key inflammatory cytokine, vigorously induces CD146 expression on the endothelium [16]. They validated that TNF-α augments sCD146 secretion from endothelial cells which facilitates leucocytes trans-endothelial migration and propagation toward inflammatory sites. Thus, in addition to mediating endothelial integrity and vascular permeability, CD146 also mediates cell recruitment and trafficking. 

Besides, Mehta et al. showed that CD146 is a major contributor to psoriasis pathogenesis [69]. In fact, IL-17A, which is mainly produced by Th17 cells, drives inflammatory reactions and intensifies psoriatic lesions. The authors distinguished a unique population of CD3+ CD4+ T cells which was primarily present in both the skin lesions and sera of psoriatic patients, the CD146+ Th17 cells. These CD3+CD4+CD146+ Th17 cells produced a significantly higher amount of IL-17 than CD146 negative cells. Likewise, PMA/ionomycin stimulation of cells from peripheral blood and skin lesions showed that CD146+ CD4+ and CD146+ CD8+ T cells produced much more IL-17A than the CD146- subset as demonstrated by flow cytometry and immunohistochemistry. Noteworthy, when compared to healthy subjects, circulating CD3+CD146+ T cells were found to be significantly elevated in peripheral blood of patients with various inflammatory autoimmune diseases such as sarcoidosis, inflammatory bowel disease, multiple sclerosis, connective tissue disease, and Behcet’s disease. In the same way, Gabsi et al. have shown that both sCD146 and IL-17A concentrations, as well as CD146+ Th17 cells, were significantly higher in the sera of fifty patients with systemic sclerosis (SSC) as compared to control subjects [27]. Impressively, low concentrations of sCD146 were correlated with pulmonary fibrosis, a life-threatening complication associated with the disease. A strong positive correlation was observed between sCD146 serum concentration and the abundance of CD146 + Th17 cells. Indeed, 24 h treatment of peripheral blood mononuclear cells (PBMCs) from SSC patients with sCD146 significantly augmented the number of CD146 + Th17 cells and increased the expression density of CD146 on the CD146 + Th17 subset. This result is extremely significant in inflammatory and autoimmune diseases given that these pathologies are characterized by vascular damages which will subsequently boost sCD146 levels in serum and thus contribute to a poor disease prognosis. So far, sCD146 has been demonstrated to act both on endothelial cells and leucocytes (Th17 cells) in response to inflammatory cytokines and increases the ratio of the CD146 + Th17 subset which will facilitate the patrolling of these cells on the endothelium and their subsequent extravasation. sCD146 can also exert a chemotactic effect on the recruited immune cells to guide them toward inflammatory sites, thereby exacerbating inflammatory responses. Excessive inflammatory reaction can lead to sepsis, induce coagulation factors, activate oncogenes, or permanently damage nearby tissues and organs [70]. More recently, Bardin et al. revealed that the levels of sCD146 in patients with systemic sclerosis is much higher than that in healthy subjects, 870 ± 30 ng/mL versus 495 ± 16 ng/mL. Besides, they validated the correlation existing between low levels of sCD146 and pulmonary fibrosis, a severe manifestation of the disease [71]. Indeed, in vivo experiments using non-immunocompromised CD146-deficient mice showed an increased sensitivity to develop fibrotic lesions in bleomycin-treated animals as compared to wild type mice. This effect was reversed by injecting sCD146 which protects mice from severe fibrotic lesions. Thus, CD146 appears to be an active mediator between autoimmune diseases such as systemic sclerosis and fibrotic events through signaling pathways that involve the Wnt cascade and probably others that are not defined yet. Similarly, in neurodegenerative diseases, sCD146 was elevated in the cerebrospinal fluid (CSF) of patients with active multiple sclerosis (MS), as compared to patients with inactive MS or non-demyelinating diseases [37]. The pathological increase in sCD146 in CSF was positively correlated with levels of pro-inflammatory cytokines as IL-2, IFN-γ, TNF-α, and IL-17A. Unfortunately, the abnormally elevated sCD146 levels in CSF negatively impact the blood brain barrier (BBB) integrity, increase permeability, damage vessels, and recruit inflammatory leucocytes, all factors promoting disease progression. In atherosclerosis, a process in which vessels are progressively clogged, as plaques enlarge in size, vessel’s intima thickens and oxygen supply from the arterial lumen to this area declines [72]. This creates a hypoxic state, a phenomenon that activates CD146 gene expression in addition to other inflammatory cytokines [73,74]. The newly induced inflammatory microenvironment potentiates the release of angiogenic factors that stimulate sprouting angiogenesis from the vasa vasorum [72]. Neovascularization facilitates the infiltration of macrophages into the atherosclerotic plaque. This enhances lipid deposition into the arterial wall and exacerbates inflammation which drive atherosclerosis lesion progression. In view of these results, the urge to therapeutically target CD146/sCD146 raises for the sake of easing its adverse effects in various autoimmune diseases but also other pathologies. 

### 4.4. CD146 in Cancer 

Initially described by Johnson and colleagues in 1987 as a tumor antigen expressed on advanced primary melanoma and metastatic lesions, CD146 was later found to be overexpressed in a broad range of cancer cell lines not limited to pancreatic, breast, prostate, ovarian, lung, kidney cancers, osteosarcoma, Kaposi sarcoma, angiosarcoma, glioblastoma, and leiomyosarcoma [14,20,24]. In fact, how cancer cells preferentially upregulate the adhesion molecule CD146 is not yet fully elucidated. A study by Liu et al. revealed an increase in the methylation status of the gene promoter in prostate cancer cells. The authors showed that the ATG upstream sequence of CD146 gene promoter becomes hypermethylated during malignant transformation of the cells which in part mediates CD146 expression [75]. Clinically, CD146 surface expression positively correlates with resistance to some chemotherapies. For instance, it has been shown that the 786-O renal cell carcinoma robustly increases membrane CD146 expression together with the soluble form of this protein in the culture media as the cultured cells become resistant to Sunitinib, a tyrosine kinase inhibitor [76]. These resistant cells, referred to as 786-R, showed higher metastatic and invasive potentials as compared to 786-O cells. Another study linked CD146 expression to cancer progression while others demonstrated higher expression during the vascularization phase of the tumor. Likewise, Liang et al. showed that CD146 expression conferred tamoxifen resistance to breast cancer cells and chemo-resistance to small-cell lung cancer cells [77,78]. Undoubtedly, all solid tumors rely primarily on angiogenesis to support the high demanding metabolic activity, growth, and metastasis. Of interest, most of the ligands that have been so far described in the literature to interact with membrane CD146 on cancer cells belong to a family of proangiogenic growth factors such as Netrin1, Wnt5a, VEGF-c, Wnt1, and FGF4 which induce angiogenic responses [59]. Indeed, the tumor microenvironment comprises a cocktail of inflammatory cytokines, growth factors, chemokines, matrix metalloproteinases, and leucocytes. Accordingly, it has been demonstrated that most of the tumor infiltrating lymphocytes (TILs) and tumor-associated macrophages (TAM) are in situ reprogrammed to promote tumor growth essentially by initiating angiogenesis [79,80,81]. Stalin et al. succeeded to assess sCD146 concentration in the tumor microenvironment and confirmed the potent elevation of this factor in CD146+ tumors. Indeed, their work showed that sCD146 exerts autocrine effects on cancer cells to induce the expression of mesenchymal markers such as vimentin, N-cadherin, and the transcription factors Snail and Slug, thus inducing epithelial to mesenchymal transition (EMT) beside its effect on activating cancer stem cell markers that enhance cancer metastatic potential [25]. In addition, sCD146, via its paracrine effect on tumor endothelial cells, induces the upregulation of membrane CD146, integrins, and other adhesion molecules which all together facilitate cancer cell metastasis and leucocyte transmigration [13]. Surprisingly, it was found that most of cancer cell lines producing the angiogenic cytokine VEGF also produced sCD146 almost equally to or higher than that produced by endothelial cells HUVECs. A comparative study elucidated the direct effect of sCD146 and VEGF on cancer cells of different origin and proved to induce proliferation, migration, and invasion in in vitro assays while protecting cells from apoptosis in response to endogenous oxidative stress [13]. Indeed, in vivo experiments on nude mice xenografted with Matrigel plugs containing luciferase-expressing Panc-1 cells showed that plugs that were injected with recombinant sCD146, unlike those treated with PBS, have higher fluorescence intensity at day 15 post injection, signifying enhanced cell survival in vivo. Moreover, the density of vascularization in these plugs was also increased in those ones treated with sCD146 as observed by Doppler ultrasound imaging and isolectin B4 labeling. To illustrate the paracrine effect exerted by cancer cells on the endothelium, conditioned media (CM) from different cultured cancer cells, UACC1273, Panc-1, and C81-61, were used to treat HUVECs. These media stimulated ECs proliferation, an effect totally abolished upon depleting the CM from sCD146 or VEGF using S-Endo-1 or bevacizumab, respectively. Thus, beside its pro-tumoral effects, sCD146 proved to be a pro-angiogenic molecule critically relevant in tumor angiogenesis. Furthermore, induced expression of CD146 on hepatocellular carcinoma and ovarian cancer cells significantly increased their invasive and metastatic potentials whilst the inhibition of this molecule impeded tumor spread and metastasis by enhancing apoptosis [82,83]. These effects were attributed to CD146-mediated activation of Rho GTPases which are directly linked to the induction of cancer cells invasion, proliferation, and metastasis. Likewise, CD146 expression was more prominent on osteosarcomas as compared to non-pathological osteoblasts [84]. Altogether, CD146 is regarded as a poor prognostic marker in most solid tumors, not only being pro-angiogenic, pro-tumoral, pro-survival, and pro-coagulant [85], but also conferring resistance to chemotherapies (Table 2). 

## 5. Interest of Using anti-CD146/sCD146 mAb to Block Pathological Angiogenesis 

Angiogenesis appears to be critically important for mediating physiological development and contributes to homeostasis. However, this biological phenomenon can have deleterious consequences if occurring randomly or becoming uncontrollable as is the case with most solid tumors. Thus, angiogenesis acts as a dual edge-sword, justifying an effective therapeutic intervention. To this end, several blocking and neutralizing antibodies have been developed to target membrane CD146 and its soluble form. The AA98 monoclonal anti-CD146 antibody generated in 2003 and targeting tumor-endothelial cells was among the first antibodies to be developed for therapeutic applications [99]. This antibody displays an efficient inhibitory effect on tumor growth and angiogenesis. In fact, the CD146-blocking antibody AA98 was designed to recognize CD146 on tumor vasculature with minimal reactivity for healthy tissue vasculature. Using HUVECs previously stimulated for 3 weeks with SMMC7721 hepatocellular carcinoma conditioned media as an immunogen, the authors injected these cells into BALB/c mice and finally selected the IgG2a/κ AA98 antibody. In vitro, AA98 was able to inhibit CD146+ endothelial cells proliferation in a concentration dependent manner but failed to induce similar effects on cancer cell lines, including melanoma A375, hepatocarcinoma SMMC7221, cervical Hela cells, and ovarian SKOV3. Similarly, AA98 reduced HUVECs migration by 75% as compared to those treated with an isotype-matched control IgG. In vivo, the injection of AA98 into chicken chorioallantoic membrane (CAM) robustly decreased vascularization almost by 7-folds as compared to IgG control CAMs. Likewise, AA98 reduced tumor angiogenesis in nude mice xenografted with leiomyosarcoma, pancreatic, and hepatocellular carcinoma to an extent reaching 50%, 41%, and 72%, respectively. Of remark, AA98 inhibitory effect on angiogenesis was attributed to the inhibition of NFκB activity in targeted cells which also decreased the expression of adhesion molecules as ICAM-1 and MMP-9, thus hampering cell migration and metastatic potentials. In addition, among the different antibodies targeting CD146, the ABX-MA1 antibody recognized both tumor and endothelial CD146 molecules [100]. This antibody inhibited the formation of melanoma cell spheroids in vitro but had no effect on their proliferative capabilities. However, the authors showed that, in vivo, the antibody was able to inhibit growth of melanoma xenografts in nude mice, an effect being essentially related to impaired tumor angiogenesis. In addition, tumor metastasis was reduced thanks to the inhibitory effect on MMP-2 expression, an enzyme strongly related to metastatic processes. Unfortunately, as AA98 and ABX-MA1 do not specifically target the tumor CD146 molecule, these antibodies seem to alter normal vascularization beside the inhibitory effect on tumor vascularization. Therefore, it remained important to develop an antibody that specifically target cancer-CD146 while maintaining minimal reactivity to endothelial CD146. This approach would allow maintaining vascular integrity while specifically blocking pathological CD146 expression on cancer cells. Our team successfully generated a tumor-specific anti-CD146 (TsCD146) monoclonal antibody [20]. Via immunofluorescence and flow cytometry experiments, TsCD146 mAb was validated to target CD146+ tumor cells while failing or only faintly labeling endothelial cells, HUVECs and HMEC-1, and smooth muscle cells HUA-SMC. Of interest, TsCD146 mAb reduced CD146+ tumor cells proliferation as well as CD146 surface expression by internalizing the molecule. Indeed, animal model of xenograft revealed that TsCD146 mAb diminished CD146+ tumors growth by inhibiting proliferation while inducing apoptosis. Imaging validated that this mAb specifically detects CD146+ tumors both in vivo and in human biopsies. Furthermore, it is able to detect cancer CD146+ microparticles in sera from patients, making it an excellent biomarker for the diagnosis and early detection of human CD146+ cancers for personalized therapy. 

Noteworthy, sCD146 has also been regarded as a poor prognostic factor in nearly all solid tumors and most autoimmune diseases. In fact, sCD146 has been detected in the sera of cancer patients but also in the interstitial fluid of patients with autoimmune diseases and chronic inflammation. Besides, we have recently shown that sCD146 is associated with pro-thrombotic events in cancer patients, which elevate the risk of thromboembolism [25,85]. Due to these devastating adverse effects imposed by sCD146 in tumors, our team generated an anti-soluble CD146 antibody referred to as M2J-1. This neutralizing anti-sCD146 mAb attested its therapeutic efficacy by inhibiting tumor growth, angiogenesis and thromboembolism in xenografted nude mice with either human melanoma cells or pancreatic carcinoma. Moreover, it enhances cancer cells’ susceptibility to undergo apoptosis in vivo. Importantly, M2J-1 mAb abolished tumor-induced endothelial cell proliferation and vascularization both in vivo and ex vivo as evidenced by Doppler ultrasound imaging in nude mice implanted with Matrigel plugs pre-conditioned with either irrelevant IgG or M2J-1. Of remark, a key feature of this antibody relies in its ability to interact with sCD146 while maintaining no/low affinity to bind membrane CD146, thus limiting the adverse effects seen with other anti-CD146 antibodies that can interact with any cell expressing this protein.

**Figure 2:**
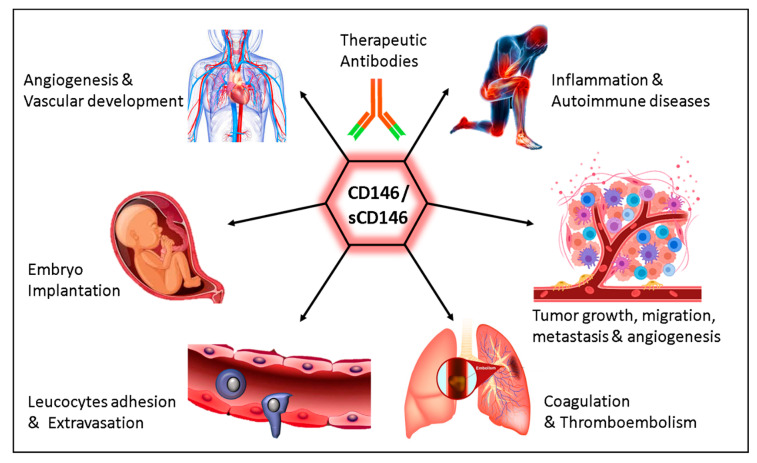
Illustration summarizing the multifaceted role of CD146/sCD146 in physiology and pathology.

Thus, in view of the major role of CD146/sCD146 in numerous pathologies related to angiogenesis, different anti-CD146/sCD146 antibodies have been generated that are currently tested for diagnostic purposes but also for therapeutic objectives (Figure 2). These newly generated anti-CD146/sCD146 antibodies will pave the way for a better targeted therapy and more relevant personalized treatment in the context of angiogenic diseases.

## Figures and Tables

**Table 1:** CD146 binding partners and ligands. A summary of proteins validated to interact with membrane CD146 and the consequent biological effects.

	Interaction	Type of Interaction	Biological Significance	References
**CD146**	VEGF-c	Ligand	Lymphatic system development	[38]
	FGF-4	Ligand	Regulate morphogenesis/Mediate cellular polarity	[39]
	Netrin-1	Ligand	Enhance VEGF-a signaling/Pro-angiogenic	[32]
	Wnt-1	Ligand	Induce fibroblasts activation and proliferation	[40]
	Wnt-5a	Ligand	Cytoskeleton remodeling/Increase cell migration	[41]
	VEGFR-2	Co-receptor	Enhance VEGFR2 signaling/Pro-angiogenic	[11]
	PDGFR-β	Co-receptor	Pericytes recruitment/Vessel stabilization	[36]
	Laminin-421	Ligand	Cancer metastasis/Retina development	[42]
	Laminin-411	Ligand	Lymphocytes extravasation in to CNS	[43]
	Galectin-1	Ligand	Endothelial cells survival/Vascular development	[33]
	Galectin-3	Ligand	Cancer progression and metastasis	[44]
	S100-A8/A9	Ligand	Chemotactic effect on cancer cells/Pro-metastatic	[45]
	Matriptase	Ligand	Stimulate neuron differentiation/Cancer invasion	[46]

**Table 2:** Biological consequences of CD146 expression on various cancer cells. Cancers are grouped according to their classification; genito-urinary and gynecologic (yellow), gastrointestinal (blue), and skin, bone, breast, and lungs (Green).

	Type of Cancer	Biological Consequences	References
**CD146/sCD146**	Cervical	Increase cell migration/Tumor metastasis	[86]
	Endometrial	Tumor angiogenesis/Tumor metastasis	[86]
	Ovarian	Cell survival/Tumor metastasis/Poor prognosis	[87,88]
	Prostate	Increase cell invasiveness/Tumor metastasis	[75,89]
	Renal cell carcinoma	Tumor recurrence/Chemotherapy resistance	[76,90]
	Breast	Increase cell migration/Induce EMT/Chemotherapy resistance	[91,92]
	Melanoma	Cell survival/Tumor angiogenesis/Tumor metastasis	[14,93]
	Non-small cell Lung cancer	Poor prognosis/Poor overall survival rate	[93,94]
	Osteosarcoma	Tumor metastasis/Poor prognosis	[95]
	Colon	Immune escape/Induce EMT	[96]
	Gastric	Induce EMT/Tumor metastasis	[97]
	Gallbladder adenocarcinoma	Increase cell invasiveness/Tumor metastasis/Poor prognosis	[98]
	Hepatocellular carcinoma	Increase cell invasiveness/Tumor metastasis/Poor prognosis	[83]
	Pancreatic carcinoma	Tumor growth and progression/Poor prognosis	[13]
